# How CRISPR/Cas9 Gene Editing Is Revolutionizing T Cell Research

**DOI:** 10.1089/dna.2021.0579

**Published:** 2022-01-12

**Authors:** Kristoffer Haurum Johansen

**Affiliations:** ^1^ Department of Health Technology, Technical University of Denmark, Lyngby, Denmark.; ^2^ Department of Pathology, University of Cambridge, Cambridge, United Kingdom.

**Keywords:** CRISPR/Cas9, T cells, 3R, screening, gene editing

## Abstract

Clustered regularly interspaced short palindromic repeats (CRISPR)/Cas9 allows for precise gene targeting in mammalian cells, including T cells, allowing scientists to disrupt or edit specific genes of interest. This has enabled immunologists to investigate T cell functions as well as opened the path for novel therapeutics involving gene editing of T cells *ex vivo* before transferring these back to patients to increase T cell efficacy. This review outlines how CRISPR/Cas9 has transformed T cell research allowing immunologists to rapidly probe the roles of genes in T cells thus paving the way for novel therapeutics. Furthermore, this review describes how these tools reduce the requirement for genetic mouse models, while increasing the translational potential of T cell research.

## Introduction

Clustered regularly interspaced short palindromic repeats (CRISPR)/Cas9 has revolutionized the way scientists approach research in all fields of biology and medicine. By enabling easy, swift, and precise manipulation of the genetic code, scientists can probe the roles of novel genes with an unprecedented speed and precision. The Nobel prize awarded to Jennifer Doudna and Emmanuelle Charpentier in 2020 for their discoveries of CRISPR/Cas9 celebrates the beginning of a biological revolution. CRISPR was discovered in bacteria as a defense mechanism used to disrupt invading foreign DNA from invading bacterial viruses (i.e. bacteriophages) by guiding a DNA cleaving nuclease to the foreign genomic DNA (Makarova *et al.*, 2006; Barrangou *et al.*, 2007; Garneau *et al.*, 2010). Multiple CRISPR nucleases have since been discovered that can cut the foreign DNA, with the most investigated nuclease being the single subunit type II CRISPR nuclease, Cas9. CRISPR/Cas9 is not the first or only gene editing tool, but it is adaptable to edit most sections of the genome with high precision and ease. In T cell immunology, the application of CRISPR/Cas9 has allowed scientists to investigate which genes are necessary for cancer immune evasion (Kearney *et al.*, 2018; Pan *et al.*, 2018; Lawson *et al.*, 2020), immune-mediated cancer elimination and tumor infiltration (Dong *et al.*, 2019; Wei *et al.*, 2019), infection of host cells (Park *et al.*, 2017; Li *et al.*, 2020; Zhu *et al.*, 2021), development of immune cell subsets and functions of the subsets (Shifrut *et al.*, 2018; Cortez *et al.*, 2020), as well as allowed for genetic engineering of highly efficient chimeric antigen receptor T cells (CAR-T) for fighting cancers (Rupp *et al.*, 2017). This review describes how CRISPR/Cas9 is catalyzing research in T cells, and how it is not just speeding up discoveries, but also aiding translational T cell research.

## T Cell Research and Translational Potential

T cells are critical to adaptive immune reactions where they are required for fighting pathogens and cancers. When dysregulated, T cells can be responsible for autoimmune disorders, such as multiple sclerosis or rheumatoid arthritis where aberrant autoreactive T cells disrupt tissue homeostasis. Harnessing and regulating T cells has, therefore, for decades been sought as a possible path to targeting cancers, infections, and autoimmunity. Consequently, numerous therapies have successfully targeted T cell activity, including cancer immunotherapies (Waldman *et al.*, 2020). However, T cells are highly diverse in their functions and their regulation is complex.

**FIG. 1.**
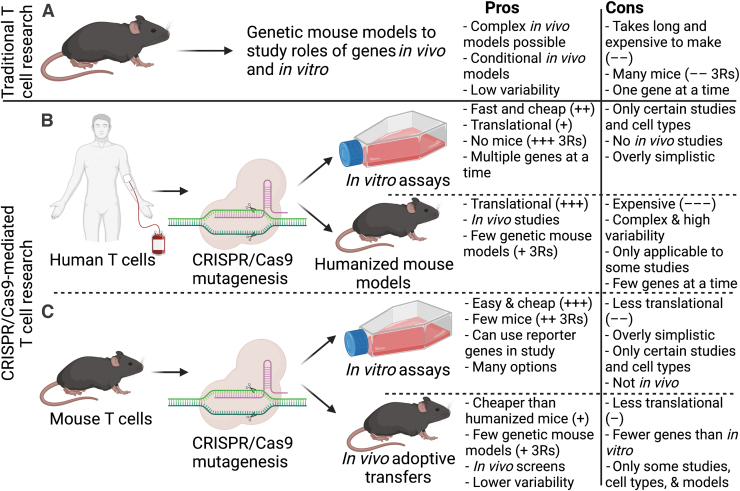
CRISPR/Cas9 as a new tool in the T cell research toolbox. Diagram of T cell research techniques using traditional genetic mouse models **(A)**, CRISPR/Cas9 in human T cells *in vitro* and in humanized mouse models **(B)**, and CRISPR/Cas9 in mouse T cells *in vitro* and followed by *in vivo* adoptive transfers **(C)**. Pros and cons illustrate considerations for using one system versus the others with + and − illustrating the extent to which one system is cheaper, more/less translational, and potential to reduce need for mice, for example, require more/less mice compared with other systems. Created with BioRender.com. CRISPR, clustered regularly interspaced short palindromic repeats.

Traditionally, T cell studies have relied on genetically modified mouse models where the gene of interest has either been knocked out, knocked in, or deleted by conditional Cre-lox-mediated recombination where the targeted gene is deleted in specific cellular subsets (Fig. 1A). This has allowed for evaluating the roles of genes as well as manipulation of specific genes in T cell subtypes. Mice are relatively easy and cheap to keep and can be bred under controlled pathogen-free conditions, thereby decreasing variability. These studies have enabled extensive characterization of immune and T cell functions in mice and allows for a way of probing functions of specific genes *in vivo*, including in infection models, autoimmune models, T cell development, and migration. Yet, there are caveats to these approaches. First, generation of genetic mouse models is time consuming, laborious, and expensive. Furthermore, although T cell findings in mice have to a remarkable extent translated to humans, there are notable differences, such as their development, subset composition, and expression patterns (Mestas and Hughes, 2004), and only a fraction of therapies with efficacy in mouse models successfully translate to humans in clinical trials (von Herrath and Nepom, 2005; Mak *et al.*, 2014; Tao and Reese, 2017). Consequently, there has been an increased effort in developing systems that are directly translational to human disease. One such system is CRISPR/Cas9.

## Mechanism of CRISPR/Cas9

Cas9 is guided to the foreign DNA by a guide RNA (gRNA) composed of a trans-activating CRISPR RNA (tracrRNA), which facilitates binding of the Cas9 enzyme, and a CRISPR RNA (crRNA), which engages the target DNA and the tracrRNA bound in the Cas9 enzyme, thus guiding the enzyme specifically to its target site. The gRNA-bound Cas9 enzyme binds gRNA-complementary genomic DNA sequences upstream of protospacer adjacent motifs (PAM) (Anders *et al.*, 2014), and cleaves the DNA inducing a double-stranded break (Garneau *et al.*, 2010; Anders *et al.*, 2014).

The creative discovery that initially catalyzed the applicability of CRISPR/Cas9 in common scientific research was the combination of the tracrRNA with the crRNA to create a single gRNA (sgRNA) (Jinek *et al.*, 2012). Introducing this sgRNA and the Cas9 enzyme into eukaryotic cells allowed scientists to cut the genome at any sequence upstream of a PAM motif recognized by the chosen Cas9 enzyme (NGG for *Streptococcus pyogenes* Cas9) with high specificity and efficacy (Doench *et al.*, 2016). As these PAM motifs are short and commonly found throughout the genome, sgRNAs can be designed for targeting most regions throughout the genome, thus allowing for precisely targeting most, if not all, genes. The double-stranded break is then repaired by either nonhomologous end joining (NHEJ) or homology-directed repair (HDR). NHEJ is error prone and often results in random insertions or deletions; in turn, these errors often result in frameshift mutations or deletions that knockout (KO) the targeted gene (i.e., prevent effective translation of the mRNA encoded by the gene). CRISPR/Cas9 was thus adapted as a gene KO tool in mammalian cells (Cho *et al.*, 2013; Cong *et al.*, 2013; Jinek *et al.*, 2013; Mali *et al.*, 2013). By introducing a DNA template alongside the Cas9 enzyme and sgRNA, Cas9 can also be adapted as a gene editing tool, where the template DNA is used as template for HDR-mediated repair, thereby replacing the DNA at the site of the double-stranded break (Cho *et al.*, 2013; Cong *et al.*, 2013; Jinek *et al.*, 2013; Mali *et al.*, 2013). Thereby CRISPR/Cas9 can be used to repair faulty genes as therapy, as well as for research purposes for genetic modification. Together, these tools have quickly transformed the way scientists study the genome and led to numerous tools harnessing the sequence-specific selectivity of the sgRNA/Cas9 complex.

## CRISPR/Cas9-Mediated Gene KO in T Cells

In mouse T cells, CRISPR/Cas9 mutagenesis can be performed in multiple ways. It can be done by directly introducing Cas9-encoding mRNA and sgRNAs (Mandal *et al.*, 2014; Su *et al.*, 2016) or sgRNAs coupled to Cas9 protein (Cas9 ribonuclear proteins [RNPs]) using electroporation (Seki and Rutz, 2018; Nussing *et al.*, 2020). Electroporation of such RNPs is preferred over electroporation of Cas9-encoding mRNA, as RNPs have been shown to have higher targeting efficiency in T cells (Schumann *et al.*, 2015). Alternatively, the sgRNA is introduced by γ-retroviral transduction into Cas9 transgenic mice expressing the Cas9 enzyme endogenously in T cells. γ-Retroviruses efficiently infect activated proliferating mouse T cells (Zhang *et al.*, 2003; Kerkar *et al.*, 2011), and this approach has been shown to effectively target mouse T cell genes (Huang *et al.*, 2019; Roy *et al.*, 2020). Retroviral delivery of sgRNAs has the important added benefit that retroviruses are integrated into the genome, and hence can be identified by next-generation sequencing allowing for pooled CRISPR/Cas9-mediated screening. In human T cells simple retroviral delivery of the sgRNA is not sufficient as Cas9 has to be introduced alongside the sgRNA. All-in-one lentiviral vectors that express both the sgRNAs and Cas9 exist (Sanjana *et al.*, 2014), but the viral vector size will approach the packaging limit of retroviruses (8–10 kb) (Miller, 1992) resulting in reduced transduction efficacy. A creative solution to this problem termed sgRNA lentiviral infection with Cas9 protein electroporation (SLICE), solves this by combining electroporation of Cas9 with lentiviral introduction of sgRNAs (Shifrut *et al.*, 2018). These techniques allow for effective CRISPR/Cas9-mediated screening directly in human T cells.

## How CRISPR/Cas9 Is Benefiting T Cell Research and Therapies

### CRISPR/Cas9 in T cell research

CRISPR/Cas9 allows immunologists to study the roles of genes in a high-throughput manner directly in human T cells, which will likely provide increased translational potential in preclinical studies as a supplement to genetically modified animal models (Fig. 1). CRISPR/Cas9 has the benefit of speeding up research considerably by reducing the need for generating genetic mouse models for each gene of interest with the important added benefit that fewer mice are needed, supporting the principles of the 3Rs (Reduce, Reuse, and Recycle) (Fig. 1) (Kirk, 2018).

As outlined, genes can be manipulated with CRISPR/Cas9 in both human and mouse T cells *in vitro* (Fig. 1B, C). These *in vitro* assays provide an accessible method for screening roles of genes directly in human T cells and can be designed to answer a broad range of cell biology questions without the need for mice. However, they risk being overly simplistic and do not take into account the tissue distribution of the cells as well as interactions with other cell types *in vivo*. Mouse T cells can be studied *in vivo* with CRISPR/Cas9 with relative ease by adoptive T cell transfer of Cas9-edited mouse T cells (Fig. 1C) (or by reconstitution with Cas9-edited hematopoietic stem cells (LaFleur *et al.*, 2019)). These studies provide valuable insight into tissue distribution, interplay between the cells and the tissues, biological function, and differentiation *in vivo*, and allow for investigating immunological responses to pathogens, autoimmune disease, or cancer.

CRISPR/Cas9 has already aided in discovery of multiple gene programs critical for human T cell activation, proliferation, and signaling (Shifrut *et al.*, 2018), as well as genetic circuits involved in T cell differentiation (Schumann *et al.*, 2020). One challenge of studying human T cells is that they cannot easily be studied *in vivo* after Cas9-mediated editing. To overcome this challenge, scientists could combine Cas9-modified human T cells with organoid systems, where organized three-dimensional tissue cultures are grown in culture (Dijkstra *et al.*, 2018; Yuki *et al.*, 2020), to investigate the functions of the Cas9-modified T cells *in situ* to simulate *in vivo* conditions. Nonetheless, such organoid systems so far only allow for experiments involving one tissue (and do not effectively replicate the complex interplay between tissues), and are limited to studying certain tissue types. Cas9-edited human T cells can also be studied *in vivo* in so-called humanized mice, which are immunodeficient mice that are reconstituted with human immune cells. These have shown some potential in increasing translational potential of therapies from preclinical models (Tao and Reese, 2017; Allen *et al.*, 2019), and could in combination with Cas9-modified T cells allow for studies of genetically modified human T cells in an *in vivo* setting. Unfortunately, these models are not trivial to set up and are generally not broadly available to the research community (Fig. 1B) (Allen *et al.*, 2019). Nonetheless, Cas9-mediated editing of human T cells provides a novel tool for overcoming many of the hurdles with translation of T cell research conducted in mice.

In summary, these described model systems have the advantage of being high throughput compared with conventional genetically modified mouse models. Furthermore, as Cas9-edited human T cells are somewhat easier to generate, it is now possible to conduct studies in a model with greater translational potential, while accommodating the principles of the 3Rs. These benefits of using CRISPR/Cas9 in T cell research are also applicable to many other cell types. Although it is still a challenge to effectively implement these tools with many other primary human cell types, future innovation will likely enable this.

### CRISPR/Cas9 in T cell therapies

CRISPR/Cas9 has further allowed for generation of genetically modified T cells that can be used in patients for therapies. CRISPR/Cas9 has been adapted for generating efficacious CAR-T cells for therapy, which have an artificially introduced antigen receptor (Eyquem *et al.*, 2017; Liu *et al.*, 2017; Rupp *et al.*, 2017), as well as for adoptive T cell transfers of cancer-specific T cells as therapy for cancer after KO of immune checkpoint inhibitors thereby increasing the T cell efficacy (Su *et al.*, 2016; Stadtmauer *et al.*, 2020; Fix *et al.*, 2021; Kamali *et al.*, 2021). Multiple technological advances involving CRISPR/Cas9 have markedly increased the promise of efficacious CAR-T cell therapy. By using CRISPR/Cas9 in generation of CAR-T cells, the CAR can be integrated in the T cells at the T cell receptor α loci resulting in superior CAR-T cells (Eyquem *et al.*, 2017) and simultaneously, CRISPR/Cas9 can be applied to disrupt checkpoint inhibition of the CAR-T cells by KO of PD1 (Rupp *et al.*, 2017). In conjunction, the use of the Cas12a/Cpf1 CRISPR system has proven to be superior to Cas9-mediated CAR-T cell generation, and allows for CAR-T cell knock-in while simultaneously knocking out checkpoint inhibitors (Dai *et al.*, 2019). CRISPR/Cas9-mediated editing of T cells has already shown efficacy in clinical trials (Stadtmauer *et al.*, 2020), and multiple clinical trials on CAR-T cells are ongoing (Razeghian *et al.*, 2021). Similarly, it is likely that CRISPR/Cas9 will also be applied to fix genetic defects in T cells in patients with T cell-mediated primary immunodeficiencies (defective T cell responses due to gene defects).

## Conclusions

CRISPR/Cas9 has changed the way scientists conduct research in multiple fields, including the field of immunology. In T cell research CRISPR/Cas9 mutagenesis has already been used for multiple important discoveries, and has paved the way for novel, faster, and more translational techniques that in coming years have the potential to disrupt the way T cell studies are performed. Future developments will likely further enable CRISPR/Cas9-mediated development of highly efficacious T cells for cancer therapies, including CAR-T cells and adoptive T cell transfer.

## References

[B1] Allen, T.M., Brehm, M.A., Bridges, S., Ferguson, S., Kumar, P., Mirochnitchenko, O., *et al.* (2019). Humanized immune system mouse models: progress, challenges and opportunities. Nat Immunol 20**,** 770–774. 31160798 10.1038/s41590-019-0416-z PMC7265413

[B2] Anders, C., Niewoehner, O., Duerst, A., and Jinek, M. (2014). Structural basis of PAM-dependent target DNA recognition by the Cas9 endonuclease. Nature 513**,** 569–573. 25079318 10.1038/nature13579 PMC4176945

[B3] Barrangou, R., Fremaux, C., Deveau, H., Richards, M., Boyaval, P., Moineau, S., *et al.* (2007). CRISPR provides acquired resistance against viruses in prokaryotes. Science 315**,** 1709–1712. 17379808 10.1126/science.1138140

[B4] Cho, S.W., Kim, S., Kim, J.M., and Kim, J.S. (2013). Targeted genome engineering in human cells with the Cas9 RNA-guided endonuclease. Nat Biotechnol 31**,** 230–232. 23360966 10.1038/nbt.2507

[B5] Cong, L., Ran, F.A., Cox, D., Lin, S., Barretto, R., Habib, N., *et al.* (2013). Multiplex genome engineering using CRISPR/Cas systems. Science 339**,** 819–823. 23287718 10.1126/science.1231143 PMC3795411

[B6] Cortez, J.T., Montauti, E., Shifrut, E., Gatchalian, J., Zhang, Y., Shaked, O., *et al.* (2020). CRISPR screen in regulatory T cells reveals modulators of Foxp3. Nature 582**,** 416–420. 32499641 10.1038/s41586-020-2246-4 PMC7305989

[B7] Dai, X., Park, J.J., Du, Y., Kim, H.R., Wang, G., Errami, Y., *et al.* (2019). One-step generation of modular CAR-T cells with AAV–Cpf1. Nat Methods 16**,** 247–254. 30804551 10.1038/s41592-019-0329-7 PMC6519746

[B8] Dijkstra, K.K., Cattaneo, C.M., Weeber, F., Chalabi, M., van de Haar, J., Fanchi, L.F., *et al.* (2018). Generation of tumor-reactive T cells by co-culture of peripheral blood lymphocytes and tumor organoids. Cell 174**,** 1586–1598.e12. 30100188 10.1016/j.cell.2018.07.009 PMC6558289

[B9] Doench, J.G., Fusi, N., Sullender, M., Hegde, M., Vaimberg, E.W., Donovan, K.F., *et al.* (2016). Optimized sgRNA design to maximize activity and minimize off-target effects of CRISPR-Cas9. Nat Biotechnol 34**,** 184–191. 26780180 10.1038/nbt.3437 PMC4744125

[B10] Dong, M.B., Wang, G., Chow, R.D., Ye, L., Zhu, L., Dai, X., *et al.* (2019). Systematic immunotherapy target discovery using genome-scale in vivo CRISPR screens in CD8 T cells. Cell 178**,** 1189–1204.e23. 31442407 10.1016/j.cell.2019.07.044 PMC6719679

[B11] Eyquem, J., Mansilla-Soto, J., Giavridis, T., van der Stegen, S.J., Hamieh, M., Cunanan, K.M., *et al.* (2017). Targeting a CAR to the TRAC locus with CRISPR/Cas9 enhances tumour rejection. Nature 543**,** 113–117. 28225754 10.1038/nature21405 PMC5558614

[B12] Fix, S.M., Jazaeri, A.A., and Hwu, P. (2021). Applications of CRISPR genome editing to advance the next generation of adoptive cell therapies for cancer. Cancer Discov 11**,** 560–574. 33563662 10.1158/2159-8290.CD-20-1083 PMC8193798

[B13] Garneau, J.E., Dupuis, M.E., Villion, M., Romero, D.A., Barrangou, R., Boyaval, P., *et al.* (2010). The CRISPR/Cas bacterial immune system cleaves bacteriophage and plasmid DNA. Nature 468**,** 67–71. 21048762 10.1038/nature09523

[B14] Huang, B., Johansen, K.H., and Schwartzberg, P.L. (2019). Efficient CRISPR/Cas9-mediated mutagenesis in primary murine T lymphocytes. Curr Protoc Immunol **124,** e62. 10.1002/cpim.62 PMC6340735 30312021

[B15] Jinek, M., Chylinski, K., Fonfara, I., Hauer, M., Doudna, J.A., and Charpentier, E. (2012). A programmable dual-RNA-guided DNA endonuclease in adaptive bacterial immunity. Science 337**,** 816–821. 22745249 10.1126/science.1225829 PMC6286148

[B16] Jinek, M., East, A., Cheng, A., Lin, S., Ma, E., and Doudna, J. (2013). RNA-programmed genome editing in human cells. Elife **2,** e00471. 10.7554/eLife.00471 PMC3557905 23386978

[B17] Kamali, E., Rahbarizadeh, F., Hojati, Z., and Frodin, M. (2021). CRISPR/Cas9-mediated knockout of clinically relevant alloantigenes in human primary T cells. BMC Biotechnol **21,** 9. 10.1186/s12896-020-00665-4 PMC7844963 33514392

[B18] Kearney, C.J., Vervoort, S.J., Hogg, S.J., Ramsbottom, K.M., Freeman, A.J., Lalaoui, N., *et al.* (2018). Tumor immune evasion arises through loss of TNF sensitivity. Sci Immunol **3,** eaar3451. 10.1126/sciimmunol.aar3451 29776993

[B19] Kerkar, S.P., Sanchez-Perez, L., Yang, S., Borman, Z.A., Muranski, P., Ji, Y., *et al.* (2011). Genetic engineering of murine CD8+ and CD4+ T cells for preclinical adoptive immunotherapy studies. J Immunother 34**,** 343–352. 21499127 10.1097/CJI.0b013e3182187600 PMC3100770

[B20] Kirk, R.G.W. (2018). Recovering the principles of humane experimental technique: the 3Rs and the human essence of animal research. Sci Technol Human Values 43**,** 622–648. 10.1177/0162243917726579 PMC6027778 30008492

[B21] LaFleur, M.W., Nguyen, T.H., Coxe, M.A., Yates, K.B., Trombley, J.D., Weiss, S.A., *et al.* (2019). A CRISPR-Cas9 delivery system for in vivo screening of genes in the immune system. Nat Commun **10,** 1668. 10.1038/s41467-019-09656-2 PMC6458184 30971695

[B22] Lawson, K.A., Sousa, C.M., Zhang, X., Kim, E., Akthar, R., Caumanns, J.J., *et al.* (2020). Functional genomic landscape of cancer-intrinsic evasion of killing by T cells. Nature 586**,** 120–126. 32968282 10.1038/s41586-020-2746-2 PMC9014559

[B23] Li, B., Clohisey, S.M., Chia, B.S., Wang, B., Cui, A., Eisenhaure, T., *et al.* (2020). Genome-wide CRISPR screen identifies host dependency factors for influenza A virus infection. Nat Commun **11,** 164. 10.1038/s41467-019-13965-x PMC6952391 31919360

[B24] Liu, X., Zhang, Y., Cheng, C., Cheng, A.W., Zhang, X., Li, N., *et al.* (2017). CRISPR-Cas9-mediated multiplex gene editing in CAR-T cells. Cell Res 27**,** 154–157. 27910851 10.1038/cr.2016.142 PMC5223227

[B25] Mak, I.W., Evaniew, N., and Ghert, M. (2014). Lost in translation: animal models and clinical trials in cancer treatment. Am J Transl Res 6**,** 114–118. 24489990 PMC3902221

[B26] Makarova, K.S., Grishin, N.V., Shabalina, S.A., Wolf, Y.I., and Koonin, E.V. (2006). A putative RNA-interference-based immune system in prokaryotes: computational analysis of the predicted enzymatic machinery, functional analogies with eukaryotic RNAi, and hypothetical mechanisms of action. Biol Direct **1,** 7. 10.1186/1745-6150-1-7 PMC1462988 16545108

[B27] Mali, P., Yang, L., Esvelt, K.M., Aach, J., Guell, M., DiCarlo, J.E., *et al.* (2013). RNA-guided human genome engineering via Cas9. Science 339**,** 823–826. 23287722 10.1126/science.1232033 PMC3712628

[B28] Mandal, P.K., Ferreira, L.M., Collins, R., Meissner, T.B., Boutwell, C.L., Friesen, M., *et al.* (2014). Efficient ablation of genes in human hematopoietic stem and effector cells using CRISPR/Cas9. Cell Stem Cell 15**,** 643–652. 25517468 10.1016/j.stem.2014.10.004 PMC4269831

[B29] Mestas, J., and Hughes, C.C. (2004). Of mice and not men: differences between mouse and human immunology. J Immunol 172**,** 2731–2738. 14978070 10.4049/jimmunol.172.5.2731

[B30] Miller A.D. (1992). Retroviral vectors. Curr Top Microbiol Immunol 158**,** 1–24. 1582242 10.1007/978-3-642-75608-5_1

[B31] Nussing, S., House, I.G., Kearney, C.J., Chen, A.X.Y., Vervoort, S.J., Beavis, P.A., *et al.* (2020). Efficient CRISPR/Cas9 gene editing in uncultured naive mouse T cells for in vivo studies. J Immunol 204**,** 2308–2315. 32152070 10.4049/jimmunol.1901396

[B32] Pan, D., Kobayashi, A., Jiang, P., Ferrari de Andrade, L., Tay, R.E., Luoma, A.M., *et al.* (2018). A major chromatin regulator determines resistance of tumor cells to T cell-mediated killing. Science 359**,** 770–775. 29301958 10.1126/science.aao1710 PMC5953516

[B33] Park, R.J., Wang, T., Koundakjian, D., Hultquist, J.F., Lamothe-Molina, P., Monel, B., *et al.* (2017). A genome-wide CRISPR screen identifies a restricted set of HIV host dependency factors. Nat Genet 49**,** 193–203. 27992415 10.1038/ng.3741 PMC5511375

[B34] Razeghian, E., Nasution, M.K.M., Rahman, H.S., Gardanova, Z.R., Abdelbasset, W.K., Aravindhan, S., *et al.* (2021). A deep insight into CRISPR/Cas9 application in CAR-T cell-based tumor immunotherapies. Stem Cell Res Ther **12,** 428. 10.1186/s13287-021-02510-7 PMC8317439 34321099

[B35] Roy, N.H., Kim, S.H.J., Buffone, A., Blumenthal, D., Huang, B., Agarwal, S., *et al.* (2020). LFA-1 signals to promote actin polymerization and upstream migration in T cells. J Cell Sci **133,** jcs248328. 10.1242/jcs.248328 PMC7502589 32907931

[B36] Rupp, L.J., Schumann, K., Roybal, K.T., Gate, R.E., Ye, C.J., Lim, W.A., *et al.* (2017). CRISPR/Cas9-mediated PD-1 disruption enhances anti-tumor efficacy of human chimeric antigen receptor T cells. Sci Rep **7,** 737. 10.1038/s41598-017-00462-8 PMC5428439 28389661

[B37] Sanjana, N.E., Shalem, O., and Zhang, F. (2014). Improved vectors and genome-wide libraries for CRISPR screening. Nat Methods 11**,** 783–784. 25075903 10.1038/nmeth.3047 PMC4486245

[B38] Schumann, K., Lin, S., Boyer, E., Simeonov, D.R., Subramaniam, M., Gate, R.E., *et al.* (2015). Generation of knock-in primary human T cells using Cas9 ribonucleoproteins. Proc Natl Acad Sci U S A 112**,** 10437–10442. 26216948 10.1073/pnas.1512503112 PMC4547290

[B39] Schumann, K., Raju, S.S., Lauber, M., Kolb, S., Shifrut, E., Cortez, J.T., *et al.* (2020). Functional CRISPR dissection of gene networks controlling human regulatory T cell identity. Nat Immunol 21**,** 1456–1466. 32989329 10.1038/s41590-020-0784-4 PMC7577958

[B40] Seki, A., and Rutz, S. (2018). Optimized RNP transfection for highly efficient CRISPR/Cas9-mediated gene knockout in primary T cells. J Exp Med 215**,** 985–997. 29436394 10.1084/jem.20171626 PMC5839763

[B41] Shifrut, E., Carnevale, J., Tobin, V., Roth, T.L., Woo, J.M., Bui, C.T., *et al.* (2018). Genome-wide CRISPR screens in primary human T cells reveal key regulators of immune function. Cell 175**,** 1958–1971.e15. 30449619 10.1016/j.cell.2018.10.024 PMC6689405

[B42] Stadtmauer, E.A., Fraietta, J.A., Davis, M.M., Cohen, A.D., Weber, K.L., Lancaster, E., *et al.* (2020). CRISPR-engineered T cells in patients with refractory cancer. Science **367,** eaba7365. 10.1126/science.aba7365 PMC11249135 32029687

[B43] Su, S., Hu, B., Shao, J., Shen, B., Du, J., Du, Y., *et al.* (2016). CRISPR-Cas9 mediated efficient PD-1 disruption on human primary T cells from cancer patients. Sci Rep **6,** 20070. 10.1038/srep20070 PMC4730182 26818188

[B44] Tao, L., and Reese, T.A. (2017). Making mouse models that reflect human immune responses. Trends Immunol 38**,** 181–193. 28161189 10.1016/j.it.2016.12.007

[B45] von Herrath, M.G., and Nepom, G.T. (2005). Lost in translation: barriers to implementing clinical immunotherapeutics for autoimmunity. J Exp Med 202**,** 1159–1162. 16275758 10.1084/jem.20051224 PMC2213225

[B46] Waldman, A.D., Fritz, J.M., and Lenardo, M.J. (2020). A guide to cancer immunotherapy: from T cell basic science to clinical practice. Nat Rev Immunol 20**,** 651–668. 32433532 10.1038/s41577-020-0306-5 PMC7238960

[B47] Wei, J., Long, L., Zheng, W., Dhungana, Y., Lim, S.A., Guy, C., *et al.* (2019). Targeting REGNASE-1 programs long-lived effector T cells for cancer therapy. Nature 576**,** 471–476. 31827283 10.1038/s41586-019-1821-z PMC6937596

[B48] Yuki, K., Cheng, N., Nakano, M., and Kuo, C.J. (2020). Organoid models of tumor immunology. Trends Immunol 41**,** 652–664. 32654925 10.1016/j.it.2020.06.010 PMC7416500

[B49] Zhang, T., Tsang, T.C., and Harris, D.T. (2003). Efficient transduction of murine primary T cells requires a combination of high viral titer, preferred tropism, and proper timing of transduction. J Hematother Stem Cell Res 12**,** 123–130. 12662443 10.1089/152581603321210208

[B50] Zhu, Y., Feng, F., Hu, G., Wang, Y., Yu, Y., Zhu, Y., *et al.* (2021). A genome-wide CRISPR screen identifies host factors that regulate SARS-CoV-2 entry. Nat Commun **12,** 961. 10.1038/s41467-021-21213-4 PMC7878750 33574281

